# The vertebrate yolk sac: evolutionary origins and current advances in *in vitro* models

**DOI:** 10.1242/dev.205384

**Published:** 2026-04-29

**Authors:** Josephine R. Blagrove, Ana E. R. Orsi, Tereza Cindrova-Davies, Kathy K. Niakan

**Affiliations:** ^1^ Loke Centre for Trophoblast Research, Department of Physiology, Development and Neuroscience, University of Cambridge, Cambridge CB2 3EG, UK; ^2^ Wellcome Trust – Medical Research Council Stem Cell Institute, University of Cambridge, Jeffrey Cheah Biomedical Centre, Puddicombe Way, Cambridge CB2 0AW, UK; ^3^ Epigenetics Programme, Babraham Institute, Cambridge CB22 3AT, UK

**Keywords:** Embryonic development, Yolk sac, Extra-embryonic endoderm, Evolution, Stem cells

## Abstract

A key innovation in the evolutionary history of animals was the emergence of embryonic yolk, maternal nutrient deposits in the egg primarily composed of lipids and vitellogenin proteins. To contain and utilise yolk as a nutritional resource, many species have co-opted extra-embryonic tissue, named the extra-embryonic endoderm, to form a yolk sac. Extra-embryonic endoderm is believed to be conserved across vertebrate evolution, although its form and function have varied greatly over the past 500 million years. The yolk sac retains an essential role in nutrient transport, even in mammalian species that subsequently lost their yolk, and has also evolved additional functions in signalling and embryonic patterning. In this Review, we summarise our current understanding of vertebrate extra-embryonic endoderm evolution and diversity. We synthesise recent findings in reptiles to examine the evolution of the yolk sac and extra-embryonic endoderm in amniotes. Finally, we discuss how *in vitro* models can illuminate the function of this evolutionarily ancient extra-embryonic tissue, especially in the context of human development, in which yolk sac samples are extremely limited for investigation.

## Teaser

**Summary:** This Review examines the evolutionary history of the extra-embryonic endoderm, an evolutionarily ancient tissue co-opted to form the yolk sac, and explores how *in vitro* models are uncovering its biology.

## Introduction

In much of the animal kingdom, embryonic yolk is inconspicuous, despite being a key innovation in the evolution of animal embryos. Animal egg cytoplasm is rich with fats, glycogen, and yolk platelets containing vitellogenin. This precursor protein contributes to the majority of protein found in yolk, as well as some of the lipids through post-translational addition of lipid groups (Jorgensen, 2008). Many invertebrates and some vertebrates produce eggs with relatively modest yolk reserves, relying instead on rapid development and external feeding to support their growth. However, despite requiring substantial maternal investment, the convergent evolution of yolk-rich eggs has occurred independently multiple times across animal lineages (Box 1). Birds, reptiles and fish, as well as some cephalopods and arthropods, produce eggs filled with substantially more yolk, which can sustain an embryo through an extended, energy-intensive period of development (Eckelbarger and Hodgson, 2021). Furthermore, some animals have evolved alternative reproductive strategies to yolk in order to deliver greater maternal nutrients to the developing offspring, including placentation, in which the mother provides continuous nutrient supply to the embryo through specialised tissues for the whole period of gestation.

Box 1. Convergent evolution of the animal yolk sac The presence of yolk in the metazoan embryo likely predates the Cambrian explosion (∼530 million years ago), given evidence from Ediacaran fossils (∼635 to 535 million years ago) (Chen et al., 2009), and the discovery of vitellogenin homologues in cnidarians (Jorgensen, 2008). Yolk might have evolved in these ancestral metazoans to enable rapid embryonic development of complex multicellular systems (Cavalier-Smith, 2017). Yolk quantity differs vastly between animal species, and yolk sac or extra-embryonic endoderm development is not ubiquitous.

Large yolk deposits and yolk sacs are only found in bilaterians and are documented extensively in vertebrates. Yolk sac-like structures are also found in cephalopods with yolky eggs (Lee et al., 2009; Nödl et al., 2016; Montague et al., 2021). *Octopus bimaculoides* and *Octopus vulgaris* have both an internal yolk sac associated with the embryonic gut, and an external yolk sac formed through epiboly (Deryckere et al., 2020; Ibarra-García et al., 2018). The external yolk sac is vascular and muscular, and peristaltic contractions of the yolk sac initiate blood circulation into the embryo until the embryonic heartbeat becomes established.

Yolk sacs have recently been discovered in some *Pleurocryptella* isopod crustaceans; however, it is currently unclear whether this tissue is embryonic or extra-embryonic (Williams et al., 2024).

Some insects also produce very yolky embryos, with the yolk becoming internalised during development; however, viviparous insects, such as aphids, do not (Ogawa and Miura., 2014). Extra-embryonic tissue forms around the cellular blastoderm stage, and becomes closely associated with the yolk. These are termed serosa and amnion in most insects, or the amnioserosa in some flies, including *Drosophila*. The insect yolk sac refers to the cortical layer of the yolk, rather than a true cellular layer, but contains syncytial nuclei throughout (Campos-Ortega and Hartenstein, 1997; Panfilio and Chuva de Sousa Lopes, 2022; Schmidt-Ott and Kwan, 2022).
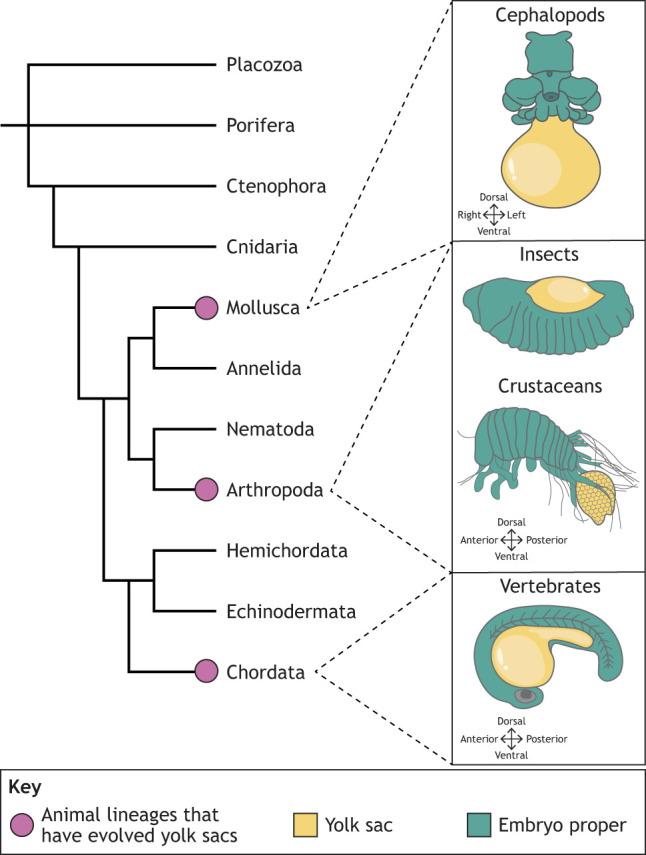

In vertebrates that do produce yolky eggs, a tissue called **extra-embryonic endoderm** (see Glossary, Table 1) has evolved to support yolk function as a nutritional source. The extra-embryonic endoderm, often alongside **extra-embryonic mesoderm**, forms the yolk sac, which envelops the yolk mass and mediates nutrient uptake into the developing embryo. Along with the **definitive endoderm**, extra-embryonic endoderm can also contribute to embryonic tissues, such as the gut (Batki et al., 2024; Kwon et al., 2008; Nowotschin et al., 2019; Rothová et al., 2022; Viotti et al., 2014). Although most complex yolk sacs also contain layers of mesoderm, which primarily function to vascularise the yolk sac, this Review focuses on extra-embryonic endoderm. The cellular yolk sac and its associated mesodermal tissues have evolved multiple times, but the role of extra-embryonic endoderm in yolk storage is conserved across most vertebrates. The evolution of extra-embryonic mesoderm has been extensively reviewed recently by Nehme et al. (2025).

**Table 1.** Glossary

Term	Definition
Allantois	One of the four key extra-embryonic membranes that evolved in amniotes. In reptiles and birds, the allantois is an extra-embryonic membrane that forms a sac used for gas exchange and storage of waste products from the embryo. In placental mammals, the allantois fuses with the chorion to form the chorioallantoic placenta and contributes to the umbilical cord.
Anterior visceral endoderm (AVE) (mammals only)	A region of thickened cells in the visceral endoderm that expresses Nodal and Wnt antagonists, controlling the onset of gastrulation on the posterior side of the epiblast. In mouse, these cells originate from the distal visceral endoderm, which migrates upwards and anteriorly to form the AVE. This term originated in the study of mouse development but is now commonly applied to non-rodent mammals. In human, the analogous tissue is sometimes called the ‘anterior hypoblast’. However, this Review will only use the term AVE in relation to all mammals to avoid confusion. This tissue is also analogous to the chick primary hypoblast.
Chorion	One of the four key extra-embryonic membranes that evolved in amniotes, the chorion forms the outermost extra-embryonic layer. In mammals, this forms from the trophoblast and extra-embryonic mesoderm to contribute to the placenta.
Definitive endoderm	One of the three germ layers that form during gastrulation, and gives rise to the epithelial lining of organs including the gut and lungs. This is distinct from the extra-embryonic endoderm in that it contributes to the embryo proper, not the yolk sac.
Epiblast (amniotes only)	Derived from the inner cell mass of the blastocyst in mammals, or from the blastoderm in birds. The epiblast gives rise to the three germ layers of the embryo proper.
Epiboly	A morphogenetic cell movement during development, whereby a layer of cells migrates over the yolk and encloses the yolk within an outer epithelium.
Extra-embryonic endoderm	Endodermal tissue that contributes to supporting structures of the embryo, including the yolk sac. In mammals, this is derived from the primitive endoderm/hypoblast. In birds, this is derived from an outer ring of cells in the blastoderm, termed the area opaca, that enclose an inner circle that forms the embryo proper, called the area pellucida. The extra-embryonic hypoblast layer that forms from the epiblast prior to gastrulation is also a form of extra-embryonic endoderm, and ultimately contributes to the yolk sac stalk. In teleost fish, this is derived from the yolk syncytial layer.
Extra-embryonic mesoderm	A mesenchymal cell type that functions in embryonic development to support extra-embryonic tissues, including the yolk sac, chorion and amnion. This tissue is derived from embryonic mesoderm, and potentially also from the primitive endoderm or primary yolk sac in primates.
Hypoblast	The hypoblast has several meanings in relation to amniote extra-embryonic endoderm. In non-human primates and humans, hypoblast typically refers to the primitive endoderm equivalent, and its derivative tissues including the visceral endoderm equivalent. The hypoblast can also refer to the primary or secondary hypoblast of chick embryos. In this Review, the term hypoblast will only be used to refer to tissue analogous to the rodent AVE in chick, and to the human primitive endoderm. In this Review, the human visceral endoderm and ‘anterior endoderm’ (AVE equivalent) will not be referred to as hypoblast, to avoid confusion. Human hypoblast derivatives will be named using mouse equivalents (visceral endoderm and AVE).
Parietal endoderm (mammals only)	Extra-embryonic endoderm that forms a simple epithelium that lines the trophoblast cavity and connects to the visceral endoderm.
Primary hypoblast (birds only)	A transient layer of extra-embryonic endodermal cells that form from polyingression of the epiblast prior to gastrulation. These cells have similar gene expression to the mammalian anterior visceral endoderm. This may be referred to as the ‘hypoblast’ in this Review.
Primitive streak	The first structure that forms at the beginning of gastrulation. Thickening of the posterior regions of the epiblast marks the primitive streak, through which the three germ layers will begin to form.
Primitive endoderm (mammals only)	The first lineage of extra-embryonic endodermal cells in mammals. These derive from the inner cell mass of the blastocyst. This term is typically used in mouse, and the equivalent tissue in human is often called ‘hypoblast’. Note ‘hypoblast’ in human can also refer to primitive endoderm derivatives, including the visceral endoderm and AVE.
Visceral endoderm (mammals only)	Extra-embryonic endoderm that lines the epiblast. In mice, visceral endoderm lines both the epiblast and the extra-embryonic ectoderm (tissue derived from the polar trophectoderm and goes on to contribute to the placenta. Depending on which tissue it lines, the mouse visceral endoderm is referred to as either embryonic or extra-embryonic, respectively. In human, the visceral endoderm is often termed ‘hypoblast’. In this Review, the term visceral endoderm will be used for both human and mouse to avoid confusion.

Vertebrates have evolved numerous other extra-embryonic tissues that support the embryo but do not contribute to the embryo proper, for instance the amnion in amniotes (reptiles, birds and mammals), which protects the embryo against mechanical shocks, provides an aqueous environment for development on land and played an important role in the evolution of terrestrial life (Starck et al., 2021), and the placenta, which transfers nutrients from mother to offspring and has evolved independently over 150 times across vertebrates (Whittington et al., 2022). However, extra-embryonic endoderm is the most ancient and well conserved, and is even conserved in the placental mammals that lost their yolk (Burton et al., 2025). Unlike the placenta, new evidence suggests that extra-embryonic endoderm could be an ancestral extra-embryonic tissue across metazoa, with secondary loss in only some clades, such as amphibians (Takeuchi et al., 2009; Cattell et al., 2012).

Evolutionarily, the initial purpose of the yolk sac was likely for the storage and transportation of increased deposits of maternal yolk in the embryo (Cindrova-Davies et al., 2017; Riddle and Hu, 2021). However, it is not limited to just this function in many species. There is emerging evidence of nutrient processing, including gluconeogenesis, in the yolk sac of fish and birds (Shibata et al., 2023a; Furukawa et al., 2024; Shimizu et al., 2024). The yolk sac wall is also the site of primitive haematopoiesis in mammals and birds through the formation of blood islands – clusters of primitive blood cells and endothelial cells (Jordan, 1910; Sabin, 1920; Takashina, 1989; Palis and Yoder, 2001; Sheng, 2010). During the process of primitive haematopoiesis, the yolk sac takes on a transitory role controlling blood coagulation, detoxification and the formation of the first blood cells, functions later taken on by the fetal liver, kidney and bone marrow after organogenesis (Cindrova-Davies et al., 2017; Goh et al., 2023). Finally, the yolk sac is a key regulator of gastrulation and formation of the body axis in mammals and birds, through tissues called the **anterior visceral endoderm (AVE)** and **hypoblast**, respectively (Stern and Downs, 2012; Thowfeequ et al., 2025). In mammals and birds, the AVE/hypoblast controls the timing and positioning of **primitive streak** formation, leading to the initiation of gastrulation occurring at the posterior side of the **epiblast**.

Despite the conserved evolutionary history of the yolk sac and its importance to the successful development of most vertebrates, it has received relatively little attention compared to other extra-embryonic tissues, such as the placenta (Burton et al., 2025). The separation of the study of mouse, human and chick embryology has led to overlapping terminology for several extra-embryonic endoderm tissues with different meanings and evolutionary relationships (see Glossary, Table 1). The study of the human yolk sac is also hindered by its transient nature and the scarcity of available samples for research. However, recent work in reptiles has shed light on the evolutionary history of both the amniote yolk sac and its signalling functions (Yoshida et al., 2016), and work in non-model anamniotes has revealed the diversity and conservation of extra-embryonic endoderm across vertebrate evolution (Takeuchi et al., 2009; Shah et al., 2024). Furthermore, advances in organoids and stem cell-based embryo models (SCBEMs) have led to an expansion of *in vitro* models of extra-embryonic endoderm and its derivative tissues. These models facilitate the investigation of the molecular biology and functional role of factors important for yolk sac development.

In this Review, we examine the evolutionary origin and developmental trajectory of extra-embryonic endoderm across vertebrates. We then explore the evolutionary origins of the AVE/hypoblast tissue in amniotes, and recent work that questions whether this is a homologous or analogous trait (i.e. whether there is common ancestry or whether this tissue evolved independently) in birds and placental mammals. Finally, we compare and contrast emerging *in vitro* models of extra-embryonic endoderm that utilise stem cells, SCBEMs and organoids. These are pertinent methods for studying the human yolk sac, a transient tissue that is particularly difficult to access but carries significant implications for our understanding of early development, pregnancy complications and fertility issues.

## The evolution and diversity of yolk sac development

### Evolution of extra-embryonic endoderm in fish

Anamniotes generally have relatively simple extra-embryonic tissues compared to amniotes. Amniotes have evolved several extra-embryonic tissues to adapt to embryonic development on land. This includes the allantoic membrane (**allantois**), which is a sac-like tissue that collects waste products produced by the embryo, and the amniotic membrane, which encompasses the embryo and is filled with fluid to maintain an aqueous environment. However, all vertebrate clades use yolk as either the sole or a major source of nutrition during embryo development. The yolk in most vertebrates is encompassed in a yolk sac, which is used to deliver nutrients from the yolk to the developing embryo. The yolk sac, or more specifically the extra-embryonic endoderm, has been conserved throughout vertebrate evolution, from fish to mammals, and is considered the oldest vertebrate extra-embryonic tissue (Ross and Boroviak, 2020; Shibata et al., 2023b).

#### Ancestral vertebrates

**Fig. 1.**
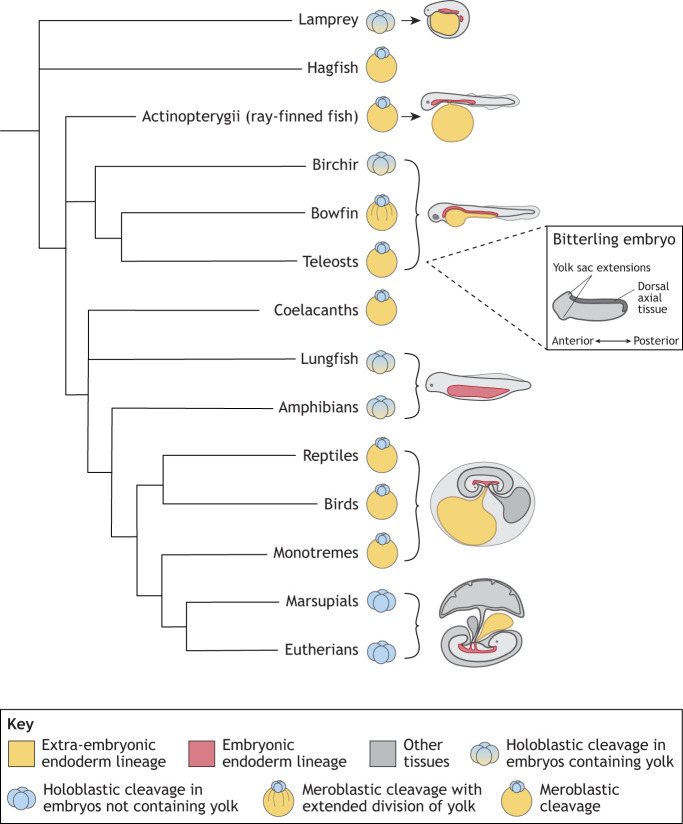
**Early cleavage patterns and yolk sac development across vertebrate species based on their phylogenetic relationship.** Extra-embryonic and embryonic endoderm are represented in yellow and red, respectively. Information on cleavage-stage embryos is adapted from Elinson (2009). Hagfish and coelacanth yolk sacs have not been sufficiently described to include diagrams here. Schematics not to scale. Branch lengths are not representative of time.

Holoblastic (complete) cleavage of the early embryo, usually in species with little yolk, is thought to be characteristic of the basal vertebrate embryo (Fig. 1) (Chea et al., 2005; Elinson, 2009). Holoblastic cleavage forms blastomeres, which can contribute to both embryonic and extra-embryonic tissues. In lamprey, one of the two most ancestral vertebrate groups (agnathans), the early embryo divides by holoblastic cleavage. The vegetal mass cells are nutritive and contain a large amount of yolk. However, these cells are not embryonic as they do not appear to contribute to any of the three germ layers (Takeuchi et al., 2009; Cattell et al., 2012) (Fig. 1). Li et al. (2019a) have shown that, later in development, lampreys develop a yolk sac. Fossils of Palaeozoic lampreys are also thought to carry a yolk sac (Miyashita et al., 2021). Extant hagfish, the other clade of the ancestral agnathans, undergo meroblastic cleavage, in which cells are incompletely cleaved because the cleavage furrow does not pass entirely through the yolky vegetal pole of the embryo. Hagfish also develop a yolk sac, but this has not been well described (Ota and Kuratani, 2008) (Fig. 1).

#### Cartilaginous fish

The main subgroup of Chondrichthyes (cartilaginous fish) is the elasmobranchs (sharks and rays). Shark and ray embryos are, in some ways, more comparable to the chick than to other fish because they have large eggs comprising a very large yolk on which an embryo develops by meroblastic cleavage (Fig. 1) (Elinson, 2009). Elasmobranch embryos are contained in a tough collagen-based egg case, which contains a large mass of egg jelly (Musa et al., 2018).

During the start of yolk sac formation, blastomeres on the periphery of the embryo proper drop down into the yolk to form a yolk syncytial layer (YSL), a multinuclear layer of cytoplasm surrounding the yolk mass (Hamlett and Wourms, 1984; Hamlett et al., 1987). The nuclei of the YSL migrate to cover the entirety of the yolk cell during epiboly. Following gastrulation, ectoderm, mesoderm and endoderm surround and vascularise the yolk mass and YSL, forming the complete yolk sac (Hamlett and Wourms, 1984; Hamlett et al., 1987; Kondakova et al., 2016). The shark yolk sac has a notable yolk sac stalk connecting it to the embryo proper (Fig. 1). Some viviparous sharks develop a placenta; however, these embryos often still contain yolk that is consumed by the embryo before yolk sac-placenta development is fully established (Buddle et al., 2021). The yolk sac will then closely interact with the uterine wall to facilitate nutrient exchange of maternal nutrients from uterine secretions to the embryo (Buddle et al., 2021; Whittington et al., 2022).

#### Ray-finned fish

Actinopterygii are a diverse group of ray-finned fish composed of bichirs, reedfish, sturgeons, paddlefishes, gars, bowfins and teleosts. Birchirs and sturgeons are members of the most distantly related groups of the ray-finned fish, and their embryos undergo holoblastic cleavage (Fig. 1). Takeuchi et al. (2009) demonstrated that the yolky vegetal cells of the birchir are likely extra-embryonic nutritional yolk cells. Furthermore, Shah et al. (2022) have shown that the sturgeon, which is closely related to the birchir, has similar extra-embryonic nutritional yolk cells. Gar and bowfin represent an intermediate or transitional form of meroblastic cleavage, with complete cleavage of large yolky blastomeres largely reduced, and most cleavage occurring in the animal pole of the embryo (Takeuchi et al., 2009). It has been observed that gar develop a yolk sac (Comabella et al., 2013; Shah et al., 2024). Despite these differences in early cleavage stages, all teleost clades develop extra-embryonic endoderm (Shah et al., 2024) (Fig. 1).

Teleosts represent the most species-rich clade of fishes and have the best-understood embryonic development because zebrafish (*Danio rerio*), a well-studied model organism, is a teleost. Zebrafish have a less-pronounced yolk sac compared to sharks and do not have a visible yolk sac stalk. Zebrafish rely entirely on nutrients from the yolk until development into a free-feeding larva is complete at 5 days post-fertilisation (dpf). Teleost meroblastic cleavage is highly derived from ancestral Actinopterygii and is more comparable to the greatly reduced divisions of the large yolky vegetal cells seen in the closer relatives of gars and bowfins (Shah et al., 2024). Similar to sharks, in teleosts, the YSL forms during the blastula stage from a subset of blastomeres that collapse their plasma membranes, leading to cell fusion and formation of a syncytium. These nuclei migrate to cover the entirety of the yolk cell during epiboly. However, cells of the YSL do not contribute to adult tissues (Kondakova and Bogdanova, 2021; Kondakova et al., 2023). In zebrafish, primitive haematopoiesis occurs in embryonic tissue, termed the intermediate cell mass, rather than the yolk sac (Bertrand et al., 2007). The zebrafish yolk sac is somewhat vascularised, but vascularisation is highly species dependent in teleosts. For example, some cichlids have much greater visible vascularisation of their yolk sac than zebrafish (Sutton et al., 2020).

One extreme example of yolk sac adaptation has been observed in bitterling fish embryos, which are parasites of freshwater mussels. These embryos anchor themselves within the mussel by a front-flip movement of the embryo on the yolk sac during gastrulation, during which yolk sac extensions form, allowing the fish to resist expulsion from the host (Fig. 1) (Yi et al., 2024).

### The extra-embryonic endoderm of amphibians

Amphibians are the only terrestrial animals that do not form a yolk sac to support development. Instead, they develop through holoblastic cleavage and have relatively little yolk compared to reptiles and birds, but still substantially more than most invertebrates (Fig. 1). The amphibian egg is divided into a yolk-rich vegetal pole and a yolk-poor animal pole. The vegetal pole gives rise to yolk-rich endodermal cells. A notable distinction between amphibians and amniotes, discussed later, is the presence of great quantities of intracellular yolk from the earliest cleavage stages onwards. The gross total of yolk and distribution of yolk across the vegetal and animal poles of the embryo impact both timing and mode of development and gastrulation across anurans (frogs and toads) and urodeles (salamanders) (Elinson and Beckham, 2002; Kaneda and Motoki, 2012). *Xenopus laevis*, the model organism of amphibians, has a relatively smaller egg size and quantity of yolk compared to other members of the Anura clade. In *Xenopus*, once the vegetal endodermal cells are depleted of yolk, they differentiate into gut and respiratory epithelia, thus contributing to the embryo proper (Chalmers and Slack, 2000). These cells are therefore not extra-embryonic endoderm. However, in the Puerto Rican tree frog, *Eleutherodactylus coqui*, the egg is 20 times the volume of *Xenopus* (measuring around 3.5 mm) with much more yolk, and the yolky endoderm cells, termed ‘nutritional endoderm cells’, do not contribute to the embryo proper (Buchholz et al., 2007; Elinson, 2009; Collazo and Keller, 2010). This has significant impact on the developmental biology of this species: the egg is better able to tolerate polyspermy, as is seen in the large eggs of birds, has slower divisions of the cells of the vegetal pole compared to the cells of the animal pole, and forms a blastopore lip closer to the animal pole, suggesting the vegetal cells are less actively involved in morphogenetic movements (Elinson and Beckham, 2002).

Some urodeles (salamander) species also appear to differ remarkably from *Xenopus* in their early development. The salamander *Ensatina eschscholtzii* is an exceptional example with eggs measuring around 6 mm that undergo meroblastic cleavage until around the 16-cell stage (Collazo and Keller, 2010). Both *E. coqui* and *E. eschscholtzii* provide interesting examples of the varying changes to early cleavage and the use of nutritional endoderm that amphibians have utilised to allow the egg and subsequent embryo to carry such substantial volumes of yolk.

The other main clade of the amphibians, the caecilians, is relatively understudied compared to the urodeles and anurans. However, the characterisation of one species, *Ichthyophis glutinosus,* suggests cleavage is asymmetric and resembles something close to a version of meroblastic cleavage. Many subdivisions occur at the animal pole, but a multinucleate cell mass remains at the vegetal pole of the embryo (Sarasin and Sarasin, 1887-1890; Dünker et al., 2000).

Overall, this shows the impact of the amount of yolk carried by the egg on the future developmental trajectory of the embryo, and that the variety of egg size across amphibians has led to significant and underappreciated diversity in early embryogenesis and in the evolution of extra-embryonic endoderm.

It could be assumed that because most amphibians do not develop a yolk sac, and key model species such as *Xenopus* do not develop extra-embryonic endoderm, the ancestral embryonic state of amphibians and the ancestor of tetrapods did not have extra-embryonic endoderm. However, Takeuchi et al. (2009) propose a different hypothesis. Given the evidence for extra-embryonic endoderm in lamprey, sturgeon and bichirs, they suggest that holoblastic cleavage, followed by formation of extra-embryonic nutritional yolk cells in the vegetal half of the embryo, could be ancestral to vertebrates, including the ancestors of amphibians. This model implies secondary loss in extant amphibians, meaning that well-studied amphibian development, in which all cells are embryonic, is a derived state. However, evidence for extra-embryonic endoderm in the closest tetrapod ancestor, the lungfish, is needed to corroborate this hypothesis (Kemp, 2011), as well as further investigation of how common extra-embryonic endoderm is across the breadth of the amphibian clade.

### Extra-embryonic endoderm of reptiles and birds

**Fig. 2.**
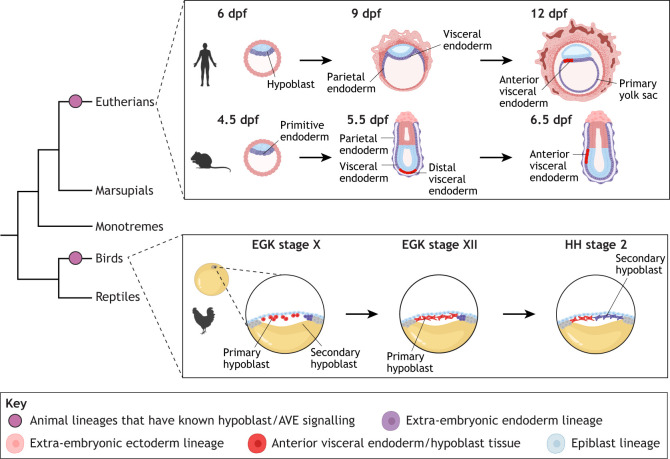
**The evolution of hypoblast signalling in the early extra-embryonic endoderm to control the temporal and spatial formation of the primitive streak.** In human and mouse embryos, the primitive yolk sac cells form during the blastocyst stage (primitive endoderm/hypoblast). After implantation into the endometrium, the primitive endoderm migrates around the inside of the trophoblast to form the yolk sac. Soon after the formation of the yolk sac, AVE/hypoblast signalling within the visceral endoderm begins to control the onset of gastrulation following its migration from the distal to visceral region. In the mouse and human, stages of development are shown in days post-fertilisation (dpf). In chick embryos, the tissue that performs comparable signalling to the mammalian AVE, known as the primary hypoblast, forms by polyingression of the epiblast. The hypoblast tissue is shifted to the anterior as secondary hypoblast, or endoblast, begins to invade the posterior portion of this cell layer. In the chick, pre-primitive streak stages of development are described in Eyal-Giladi and Kochav (EGK) stages (Eyal-Giladi and Kochav, 1976), and the primitive streak stage is described as a Hamburger and Hamilton (HH) stage (Hamburger and Hamilton, 1951). Created in BioRender by the Niakan lab 2026. https://BioRender.com/mhzhipm. This figure was sublicensed under CC-BY 4.0 terms.

Reptiles and birds undergo meroblastic cleavage on top of a large yolk deposited in the egg (Figs 1, 2). A **primitive endoderm**-like layer forms beneath the epiblast, likely through the ingression of epiblast cells to form a new layer beneath the epiblast, termed ‘polyingression’ (meaning cells ingress from multiple points of the epiblast to form the new cell layer) (Fig. 2) (Bertocchini et al., 2013). In reptiles and birds, this endodermal layer is termed the hypoblast in most literature. However, the mechanism of the early formation of the yolk sac, and whether the hypoblast contributes to the yolk sac, has not been characterised in reptiles.

By contrast, the formation of the yolk sac in the chick embryo is better understood (Nagai et al., 2015). Domestic chickens are the primary source of information on the yolk sac of non-mammalian vertebrates (Bellairs, 1953a,b; Stern and Downs, 2012; Starck, 2021). Before yolk sac formation, the yolk cell is enclosed in the yolk cell membrane, which eventually degrades. The yolk then forms direct contact with the apical yolk endoderm as the endodermal cells migrate across the yolk equator from the area opaca (the outer ring of cells in the blastoderm that enclose an inner circle that forms the embryo proper) (Sheng and Foley, 2012; Lee et al., 2025). Nagai et al. (2015) used electron microscopy to demonstrate that yolk syncytial nuclei begin to form at Eyal-Giladi and Kochav stage V and become more abundant up until post-ovipositional stages (Eyal-Giladi and Kochav, 1976). Their role in signalling or nutritional exchange has not been investigated. Formation of the bird yolk sac is characterised by initial outgrowth of the endoderm and ectoderm around the yolk, termed the ‘area vitellina’, followed by invasion of the area vitellina by the mesoderm. These three layers form a vascularised structure termed the ‘area vasculosa’, forming the yolk sac proper, and enclose the liquid yolk that the embryo will use throughout embryonic development. At the end of embryonic development, the yolk sac is withdrawn into the gut and absorbed.

A liquid yolk mass surrounded by a vascularised membrane similar to the area vasculosa was assumed to be present in all birds and reptiles. However, the corn snake (*Pantherophis guttatus*), Eastern fence lizard (*Sceloporus undulatus*), California kingsnake (*Lampropeltis getula*) (Elinson et al., 2014), snapping turtle (*Chelydra serpentina*) (Agassiz, 1857) and Italian wall lizard (*Podarcis sicula*) (Romano et al., 2002) all develop highly cellularised yolk. Despite this difference, the general structure and relationship between the amnion, **chorion**, yolk sac and allantois are very similar to birds.

Elinson et al. (2014) showed that in the late stages of development of the corn snake, the liquid yolk becomes entirely cellularised within endodermal cells and is then invaded by blood vessels from the outer yolk sac. The endodermal cells organise into monolayers around the vessels to form a ‘spaghetti-band’ structure. The endodermal cells phagocytose and likely process the yolk (Starck et al., 2021). Elinson et al. (2014) raised developmental questions that still remain regarding cellularisation of reptilian yolk, including how the cell cycle of extra-embryonic endoderm is regulated in reptiles compared to birds, whether there is initially a syncytium akin to birds or fish that becomes cellularised later in development, and what stimulates invasion by blood vessels. Although it is not definitive, Elinson et al. (2014) suggested that the cellularised yolk sac is an ancestral trait of reptiles, and the retention of liquid yolk in birds is a derived trait.

Meroblastic cleavage together with a large yolk is thought to be an ancestral amniote trait. Elinson et al. (2014) proposed that following the evolution of larger, yolkier endodermal cells in the amniote ancestor, yolk-rich cell clusters were invaded by mesoderm, which formed blood vessels for nutrient transport and gas exchange. This would enable even greater storage of yolk in the egg, and would eventually enable the transition to meroblastic cleavage, were yolk is initially not cellularised, but later yolk nutrients can be accessed by vascularisation of the yolk sac. This feature would be of great advantage to the ancestral amniote, because the embryo would have greater nutrient access and, therefore, higher chances of survival, as well as the ability to develop larger, more resource-intensive structures. However, a cellularised yolk in ancestral reptiles has not been confirmed. Therefore, this hypothesis remains speculative.

To explain this derived trait of liquid yolk in chicks, Elinson suggests their faster hatching time compared to reptiles would favour a liquid yolk (Elinson et al., 2014). During rapid development, there would be less time for cell division, meaning less yolk would be accessed despite increased energy expenditure in cellularising the yolk. It is, therefore, likely more energy efficient to keep the yolk liquid and uncellularised. Most birds take <20 days to hatch, with a few exceptions of large birds, such as the Laysan Albatross (*Phoebastria immutabilis*) at ∼66 days, and the maximum of ∼80 days in the megapode family (Megapodiidae). In contrast, snakes take ∼30 days, lizards ∼60 and crocodiles upwards of ∼100 days to develop before hatching. It is unclear whether the yolk sacs of modern-day birds arose before or after the divergence of birds from non-avian dinosaurs (Blackburn, 2021).

Both the bird and reptile yolk sacs are hematopoietic tissues. The yolk sac is the first site of haematopoiesis in the European pond turtle (Vasse and Beaupain, 1981). In chicks, haematopoiesis has been observed at Hamburger and Hamilton stage 7 of embryonic development and continues late into development up to Hamburger and Hamilton stage 45 (Guedes et al., 2014; Nagai et al., 2018; Hamburger and Hamilton, 1951).

### The yolk sac of mammals

In mammals, the yolk sac persists as a key extra-embryonic structure despite the progressive reduction of yolk in embryos across the three clades: monotremes, marsupials and eutherians (Fig. 1).

#### Monotremes

Monotremes, such as the platypus and echidna, are the only mammals to lay eggs and undergo meroblastic cleavage, reminiscent of reptilian and avian embryogenesis. The yolk sac is also functionally similar to that of reptiles and birds, forming a vascularised choriovitelline membrane (fusion of the chorion and yolk sac) for nutrient and oxygen exchange (Griffiths, 1978; Selwood and Johnson, 2006). However, the amount of yolk deposited is small compared to reptiles and birds, possibly due to the short developmental period of the embryo *in ovo*. There is also some suggestion that, during the first two-thirds of development *in ovo*, the embryo also receives nutrients and oxygen through uterine secretions exchanged through the yolk sac and chorion, before the yolk sac circulation is established (Whittington et al., 2022). The monotreme hatches at a stage comparable to the early fetal stage of eutherians. After hatching, the neonate relies on maternal lactation for continued development, thereby reducing the need for excessive yolk.

The formation of the monotreme yolk sac is not well characterised, but it is understood that the embryo forms a bilaminar layer of primitive endoderm and epiblast from the initial unilaminar blastocyst. At the unilaminar stage, the blastocyst is incomplete and likely ends around the yolk equator, having not yet completed epiboly around the yolk. The primitive endoderm migrates under the trophoblast (the outer layer of the blastocyst) to form the yolk sac (Sheng, 2015).

#### Marsupials

Marsupials do not have yolk-rich eggs and, therefore, undergo holoblastic cleavage. However, the yolk sac precursor cells form similarly to monotremes, in which the primitive endoderm forms from delamination, spreads underneath the trophoblast and surrounds the blastocyst cavity (which at this stage is a complete sphere, without any yolk) (Sheng, 2015). The marsupial yolk sac is partially vascularised and in the later third of development fuses with the chorion to form a transient choriovitelline placenta (placenta formed through fusion of the yolk sac and chorion), attached to the maternal endometrium, where it transports maternal uterine secretions to the embryo (Whittington et al., 2022). Guernsey et al. (2017) used bulk RNA sequencing of the bilaminar avascular and trilaminar vascular areas of the yolk sac placenta to demonstrate overall conservation of eutherian placental genes in the tammar wallaby (*Macropus eugenii*), and that there is differentiation of function between the vascularised area of the placenta that supports respiration and gas exchange, and the avascular area that supports histotrophic uptake of nutrients from the maternal uterine secretions. The amnion and allantois remain free-floating in some species, but bandicoots, quolls and wombats all form a chorioallantoic placenta (fusion of the chorion and allantois), as well as a persistent yolk sac placenta (Ferner and Mess, 2011).

#### Eutherians

The eutherian yolk sac develops from the primitive endoderm (referred to as the hypoblast in humans) formed in the inner cell mass of the blastocyst (Fig. 2) (reviewed by Gerri et al., 2020). This polarised epithelial layer is distinct from the remaining cells of the inner cell mass, the epiblast. The blastocyst then expands and hatches from the outer glycoprotein structure, the zona pellucida, before implanting into the uterus. Overall, this process is similar across murine and primate species, although timing varies and there are some differences in transcriptional regulation. After implantation, the embryo undergoes major and rapid developmental changes to establish a nutrient and oxygen supply from the mother.

The structure and longevity of the yolk sac vary between species. In most mammals, including ungulates and primates, the yolk sac supports nutrient transport during early development. In the second trimester, the chorioallantoic placenta takes over from the yolk sac until the end of development. Rodents are an exception because they maintain a choriovitelline placenta throughout gestation (Ornoy and Miller, 2023). In rodents and lagomorphs, an inverted yolk sac placenta is common, in which the **visceral endoderm** faces outwards towards the uterine wall, rather than forming a sac-like morphology. In humans and non-human primates, the yolk sac does not fuse with the chorion. Instead, it is free-floating in the surrounding fluid-filled space termed the ‘exocoelomic cavity’ and is connected to the embryo via the vitelline duct, which connects the yolk sac to the midgut. The free-floating yolk sac of primates is also found in some species of afrotherians and bats (Cindrova-Davies et al., 2017). Box 2 discusses the diversity of yolk sac formation found within the primate clade.

Box 2. Variation in yolk sac morphogenesis across primate clades Primate yolk sac development follows a two-phase pattern that appears broadly conserved across the clade, but varies in timing, implantation context, and structural features between lineages. In all primates studied, a transient primary yolk sac arises shortly after implantation and is subsequently replaced by a secondary yolk sac, which serves as the definitive nutritional and circulatory interface during early gestation. However, morphogenetic details and associated extra-embryonic mesoderm dynamics differ between great apes, Old World monkeys, and New World monkeys.

In haplorhine primates (apes, Old World and New World monkeys), primitive endoderm expands and migrates to form the primary yolk sac. This collapses or segregates to form the secondary yolk sac beneath the epiblast during gastrulation, which becomes the main site of nutrient uptake and haematopoiesis in early development (Ross and Boroviak, 2020).

Great apes undergo deep (interstitial) implantation in the uterine tissue, with a prominent extra-embryonic mesodermal meshwork between the primary yolk sac and trophoblast prior to its collapse. However, Old and New World monkeys implant superficially with less-extensive mesodermal meshwork in the blastocyst cavity, reflecting differences in how the primary yolk sac reorganises and in the timing of extra-embryonic mesoderm emergence. In humans and great apes, early extra-embryonic mesoderm appears before gastrulation and may partly derive from delamination of primitive endoderm-derived cells, a feature that is less extensive in some monkey species. Across primates, the secondary yolk sac shows well-developed vasculature connected to the embryo through the vitelline duct, indicating a conserved role in early circulation and nutrient exchange across haplorhines (Luckett, 1978; Ross and Boroviak, 2020).

Although fewer descriptions exist for strepsirrhine primate embryos (lemurs and lorises), evidence suggests variation in yolk sac and placental relationships (e.g. extended function of yolk sac placenta in some strepsirrhines) compared to haplorhines, reflecting deeper evolutionary divergence in reproductive strategies.

After implantation, the hypoblast (primitive endoderm) cells spread along the inner surface of the trophectoderm, lining the blastocyst cavity, becoming subdivided into visceral endoderm and **parietal endoderm**, and forms the primary yolk sac around 9-10 dpf in humans (Fig. 2) (Ross and Boroviak, 2020). Soon after, extra-embryonic mesoderm develops between this lining and the trophectoderm. From this point, up to 14 dpf, the visceral endoderm plays a role in establishing the anterior-posterior (A–P) axis of the epiblast during gastrulation, discussed in the following section (Thowfeequ et al., 2025). Between 12 and 15 dpf, the majority of the primary yolk sac fragments into small vesicles, while the portion adjacent to the epiblast reorganises into a smaller secondary yolk sac (Ross and Boroviak, 2020). The exact mechanism driving this transition is unknown, although it may reflect the separation of extra-embryonic hypoblast from definitive endoderm formed during gastrulation (Burton et al., 2025). Concurrently, the extra-embryonic mesoderm splits into two layers, one lining the trophectoderm (forming the chorion) and the other covering the secondary yolk sac, creating the fluid-filled extra-embryonic coelom between them. At this point, secondary yolk sac comprises outer mesothelium (an epithelial layer formed from mesoderm), a small middle mesodermal layer and an inner layer of endodermal epithelium.

The purpose of the primate primary yolk sac is unknown, but several hypotheses have been reviewed by Ross and Boroviak (2020). It may act as a partial source of extra-embryonic mesoderm, which forms the inner mesodermal lining of the exocoelomic cavity, which would then be supplemented with embryonic mesoderm after gastrulation. In great apes, it is also hypothesised that the primary yolk sac may provide structural support for the re-expansion of the blastocyst after implantation, because blastocyst expansion is reduced during implantation into the endometrial epithelium to invade the tissue deeply. However, this does not explain the function of the primary yolk sac in non-ape primates, which invade the endometrium in a shallower manner (Box 2).

Although the human yolk sac has historically been considered vestigial (Mossman, 1987), the yolk sac endoderm likely plays a central role in mediating nutrient transfer before the placental circulation is fully established. Although the early trophoblast can internalise uterine gland secretions, the trophoblastic villi at this stage are only sparsely vascularised. In contrast, the secondary yolk sac possesses a comparatively well-developed vascular network (Jones, 1997). Across mammalian species, yolk sac vascularisation precedes that of the allantois, and the yolk sac vessels therefore function as the earliest pathway for nutrient and gas exchange, a pattern that appears to apply to humans as well (Mossman, 1987; Wooding and Flint, 1994).

Early placental villi contain fluid-filled stromal channels that are continuous with the mesenchyme lining the extra-embryonic coelomic cavity at the chorionic plate. This suggests that substances within the villi can diffuse into the coelomic fluid, in which the secondary yolk sac is suspended (Castellucci and Kaufmann, 1982). The internal endodermal epithelium and external mesothelium of the yolk sac both exhibit ultrastructural features characteristic of absorptive tissues (Jones, 1997). A proposed histotrophic pathway during early pregnancy is therefore as follows: uterine gland secretions, supplemented by maternal serum transudate from the maternal spiral arteries, enter the intervillous space and are taken up by the trophoblast. These materials may be partially processed within trophoblastic cells and then transferred into the villous mesenchyme. From there, nutrients can diffuse along stromal channels into the coelomic fluid, where the yolk sac endoderm absorbs them. The absorbed nutrients are subsequently delivered to the embryo through the vitelline circulation (Gulbis et al., 1998; Burton et al., 2002; Cindrova-Davies et al., 2017). Toward the end of the first trimester, direct haemotrophic (meaning to receive nutrients from the blood) placental exchange becomes established and gradually supersedes this yolk sac-mediated histotrophic mechanism.

Overall, there are some key similarities among the yolk sacs of these three mammalian clades, including cellularity, vascularisation and nutrient transport. The mouse and primate yolk sac protects primordial germ cells, after gastrulation begins, from somatic signalling that could impact PGC differentiation and stemness (Witschi, 1948; Chiquoine, 1954; Ross and Boroviak, 2020). This phenomenon has been suggested to also occur in marsupials (Ullmann, 1981). It is unclear whether the yolk sac is the first site of haematopoiesis in marsupials and monotremes. However, given that both clades have vascularised yolk sacs, it is feasible that the yolk sac supports primitive haematopoiesis.

## The evolution of the AVE and hypoblast

The development of the mammalian yolk sac moves through multiple progenitor cell types, from primitive endoderm (or human hypoblast) to visceral and parietal endoderm, to the fully formed yolk sac (Fig. 2) (Gerri et al., 2020; Ross and Boroviak, 2020). The discovery and molecular functions of the rodent AVE and the chick hypoblast in directing A–P patterning have been reviewed extensively (Stern and Downs, 2012; Stower and Srinivas, 2014; Thowfeequ et al., 2025). Here, we briefly describe the role of these tissues. In the mouse, the AVE forms from the distal visceral endoderm, a region of cells that becomes thickened and transcriptionally distinct from the rest of the visceral endoderm. The distal visceral endoderm migrates upwards and anteriorly to give rise to the AVE (Fig. 2). The AVE undertakes a vital role in signalling to the embryo to block precocious gastrulation and controls the posterior positioning of the primitive streak. The AVE expresses transcription factors (including Hhex and Otx2), as well as Nodal and Wnt antagonists (including Cer1, Lefty1, Dkk1 and Sfrp1) (Stower and Srinivas, 2014; Thowfeequ et al., 2025). These antagonists constrain the onset of gastrulation to the posterior side of the epiblast around Carnegie stage 5-6 (Thomas and Beddington, 1996; Rossant and Tam, 2009; Bergmann et al., 2022; Molè et al., 2021).

In chick, the **primary hypoblast** forms from polyingression of the overlying epiblast and expresses similar factors to the rodent AVE, including Dkk1 and Cer1, but not Lefty1 (there is only one Lefty gene in chick, rather than the two copies in mammals) to block precocious gastrulation (Peter, 1938; Fabian and Eyal-Giladi, 1981; Stern and Canning, 1990; Bertocchini and Stern, 2002; Stern and Downs, 2012). The primary hypoblast is displaced to the anterior by the secondary hypoblast, or endoblast, which does not express such factors, allowing gastrulation to commence at the posterior of the epiblast (Fig. 2). However, unlike the visceral endoderm, the primary hypoblast does not contribute to the YSL or yolk sac. The chick hypoblast is thought to contribute only to the yolk sac stalk (Bellairs, 1953a,b).

The mammalian AVE and avian hypoblast are incredibly similar to extra-embryonic endoderm tissues, in gene expression, particularly of Wnt and Nodal antagonists, timing of appearance during development, and their roles in primitive streak formation. Further, the chick endoblast and the non-anterior visceral endoderm that do not express such antagonists also appear highly comparable in permitting primitive streak formation and either actively or passively constricting the AVE/hypoblast to the anterior of the epiblast. However, it is unclear whether these structures are homologous or analogous, meaning whether they evolved from a shared common ancestor (a reptile), or whether they evolved independently within the avian and mammalian clades after their divergence from reptiles. The former has previously been assumed given the presence of an extra-endodermal layer similar in morphology to the chick hypoblast layer, which was assumed to control the onset of gastrulation, comparable to the mammalian AVE and chick hypoblast (Pasteels, 1957; reviewed by Stern and Downs, 2012 and Bertocchini et al., 2013).

Yoshida et al. (2016) took a step towards addressing this question. Using RNA *in situ* hybridisation in embryos of the Chinese soft-shell turtle (*Pelodiscus sinensis*) and Madagascar ground gecko (*Paroedura picta*), they reported that in these reptilian species most AVE/hypoblast genes are not expressed in the hypoblast, in particular signalling antagonists, including Cer1, Lefty, Dkk1 and Crescent, and a few transcription factors, including Otx2 in the gecko and Gsc in both species. However, there was wide expression of several transcription factors associated with the AVE/hypoblast, including Hhex in both species, Otx2 in the turtle and Lhx1 in the gecko. Further functional investigation is required to substantiate whether the hypoblast functions to suppress the start of gastrulation occurring anteriorly in these species, akin to the functioning of the AVE/hypoblast in mammalian and avian clades. Furthermore, increased sampling of species across Reptilia, beyond the Testudines and Squamata clades investigated, needs to be characterised, both through staining of tissue to visualise RNA and/or protein, and through interrogation of single-cell RNA sequencing datasets if they were to become available in the future, to support this conclusion further. Despite these remaining questions, the authors concluded that the mammalian AVE and avian hypoblast could have evolved independently to control the onset and location of gastrulation.

The same group subsequently completed the same staining on opossum embryos, a marsupial mammal (Yoshida et al., 2016). As in reptiles, they found that most genes associated with the mouse AVE were not expressed in the opossum extra-embryonic endoderm before the formation of the primitive streak, except for the transcription factor Lhx1. This suggests the AVE-like organiser may be unique to the eutherian clade of mammals. Further analysis of RNA-sequencing data from extra-embryonic lineages could be used to elucidate whether an AVE-like population of cells exists at the onset of primitive streak formation (such as data produced by Menchero et al., 2025), which could then be functionally interrogated. Investigation of other marsupials and monotremes would add clarity to this conclusion, although, of course, these species pose difficulty in accessing early embryos.

Overall, this work suggests that the mammalian AVE and chick hypoblast may not be an amniote innovation but instead a consequence of convergent evolution in placental mammals and birds, highlighting that not every innovation of the amniote egg evolved in the reptilian ancestor. Although extra-embryonic endoderm is conserved across vertebrates, according to this hypothesis the AVE/hypoblast would be analogous in avian and mammalian clades, having convergently evolved to fulfil the same purpose using almost identical gene networks. However, more thorough investigation into a greater diversity of non-placental mammals and reptiles, through both single-cell RNA sequencing and functional experiments, could confirm if this hypothesis is correct.

### The origins of extra-embryonic endoderm signalling in the anamniote yolk sac

Some work has drawn parallels to the amniote AVE/hypoblast and endodermal tissue in anamniotes, particularly in teleosts and amphibians (Stern and Downs, 2012; Carvalho and Heisenberg, 2010). These comparisons mainly stem from similarities in gene expression in the anterior embryonic endoderm, the YSL and the AVE/hypoblast. It is hypothesised that gene expression in the amniote AVE/hypoblast largely involves co-option of genes expressed in the anterior endoderm of anamniotes to control the more recent innovation of the primitive streak.

The expression of Otx2 in the amniote AVE/hypoblast has been linked to the evolution of novel enhancers that originated in the evolution of tetrapods, which were likely co-opted in initiating the expression of Otx2 in the amphibian anterior endoderm and the amniote AVE/hypoblast (Albazerchi et al., 2007; Kurokawa et al., 2010, 2012). Hhex, which is expressed in both mouse and chick AVE/hypoblast, respectively, is also expressed in the zebrafish dorsal YSL, and plays a role in the specification of anterior and dorsal cell fates (Bischof and Driever, 2004). Nodal signalling is also a major source of comparison between anamniote YSL and amniote extra-embryonic endoderm. Nodal is expressed by the mouse AVE, and Fan et al. (2007) showed that expression of *Cyclops* and *Squint* (Nodal-related genes) from the YSL induces *Squint* expression in adjacent blastomeres of the embryo proper. This is necessary for the induction of mesoderm and endoderm cell fates, similar to Nodal signalling regulation in the rodent AVE and chick hypoblast (Foley and Stern, 2001). Therefore, it seems genes expressed in the fish YSL and amphibian anterior endoderm have been co-opted in the evolution of amniote AVE/hypoblast.

Tunicates and amphioxus, the species most closely related to vertebrates, have no extra-embryonic tissue and thus no AVE/hypoblast. However, they have very similar gene regulatory networks regarding gastrulation and endoderm development, including dorsal-ventral patterning using Lefty, and gastrulation involving Nodal and Brachyury in amphioxus (Holland et al., 1995; Yu et al., 2002; Zhang et al., 2019). These observations suggest similarities in gene regulatory networks akin to comparisons previously made to the amphibian anterior endoderm. Overall, the networks co-opted in mammalian AVE and avian hypoblasts seem to have an ancient origin in the endoderm of animals.

## *In vitro* models of extra-embryonic endoderm

There have been significant advances in the last decade in SCBEMs and organoids, using embryonic stem cells (ESCs) or tissue samples. Much of the effort thus far has focused on mouse and human models. These models offer the potential to study the molecular underpinnings of embryogenesis and lineage differentiation that complement the study of human embryos, for which there are practical and ethical limits. Importantly, SCBEMs do not replace the need for direct studies of human embryos, obtained as surplus from fertility treatments or rare donations from early elective pregnancy termination. These provide a benchmark of ground-truth from which SCBEMs can be used for further molecular characterisation. Here, we summarise the current state of *in vitro* extra-embryonic endoderm cell lines, SCBEMs and organoids (Table 2).

**Table 2.** Summary of extra-embryonic endoderm models in human and mouse systems

Species	Stage of development (dpf)	2D/3D model	Reference
Human	6	2D naïve endoderm stem cells	Linneberg-Agerholm et al., 2019
		2D peri-implantation-like endoderm cells	Okubo et al., 2024
		3D blastoids	Yu et al., 2021; Liu et al., 2021; Yanagida et al., 2021; Kagawa et al., 2022; Yu et al., 2023; Karvas et al., 2023; Pedroza et al., 2023
	12-16	2D XEN-like cells	Séguin et al., 2008; Wamaitha et al., 2015; Mackinlay et al., 2021
		3D stem cell-based embryo models	Weatherbee et al., 2023; Oldak et al., 2023; Hislop et al., 2024; Ai et al., 2023; Oura et al., 2025; Pedroza et al., 2023; Liu et al., 2023; Chen et al., 2025
	Mature yolk sac	2D yolk sac haematopoiesis model	Atkins et al., 2022
		3D yolk sac-like organoids	Tamaoki et al., 2023
Mouse	3.5-4.5	3D blastoids	Rivron et al., 2018; Sozen et al., 2019; Li et al., 2019b; Jorgensen et al., 2025
	4.5	2D naïve endoderm stem cells	Anderson et al., 2017; Ohinata et al., 2022; Linneberg-Agerholm et al., 2024
	5.0-6.0	2D XEN cells (parietal endoderm models)	Fujikura et al., 2002; Kunath et al., 2005; Shimosato et al., 2007; Niakan et al., 2010, 2013; Cho et al., 2012; Wamaitha et al., 2015
		2D XEN cells (visceral endoderm models)	Kruithof-de Julio et al., 2011; Artus et al., 2012; Paca et al., 2012; Cho et al., 2012
	4.5-8.5	3D stem cell-based embryo models	Amadei et al., 2022; Tarazi et al., 2022; Ren et al., 2025
	Mature yolk sac	2D yolk sac haematopoiesis model	Atkins et al., 2022
		3D yolk sac-like organoids	Burton et al., 2025

dpf, days post-fertilisation; XEN, *in vitro* extra-embryonic endoderm.

### Stem cell-based models of preimplantation hypoblast development

Anderson et al. (2017) showed that naïve mouse ESCs differentiate into a primitive endoderm-like state labelled naïve endoderm (nEnd) upon Wnt and activin stimulation. High levels of insulin favour mouse ESC proliferation, while low insulin levels improve nEnd differentiation (Anderson et al., 2017). nEnd cells capture the remarkable plasticity of the primitive endoderm and form 3D blastocyst-like structures called blastoids that contain trophoblast, primitive endoderm and epiblast-like cells (Linneberg-Agerholm et al., 2024). Similar to the mouse, human nEnd-like cells can be derived from naïve ESCs upon activation of WNT, activin and LIF signalling (Linneberg-Agerholm et al., 2019). However, human nEnd plasticity has not yet been determined and these cells more likely reflect later stages of extra-embryonic endoderm development based on their gene expression patterns, such as loss of OCT4 (*POU5F1*) expression. Ohinata et al. (2022) reported derivation of primitive endoderm stem cells that also model aspects of the primitive endoderm in the mouse, including contribution to visceral endoderm and parietal endoderm in chimaera assays.

Moreover, Okubo et al. (2024) generated human hypoblast-like cells (nHyCs) from pluripotent stem cells (PSCs). The nHyCs have the potential to assemble with naïve human PSCs to form 3D bilaminar structures called bilaminoids that mimic human peri-implantation development, including the patterning of the A–P axis and the formation of the pro-amniotic-like cavity. By expanding the repertoire of stem cell models of the earliest stages of hypoblast (primitive endoderm) development, these models will be useful to determine the mechanisms regulating plasticity of this lineage. Chemical reprogramming of mouse ESCs has also allowed continuous modelling of embryo development from early stages to gastrulation (Ren et al., 2025).

### Stem cell-based models of peri- and post-implantation extra-embryonic endoderm development

Embryoid bodies are among the earliest models to simulate aspects of early development, including the spontaneous differentiation of extra-embryonic endoderm. Doetschman et al. (1985) developed embryoid bodies with structures similar to visceral yolk sac and blood island erythrocytes. These 3D models included inner epiblast-like cells and an outer layer of cells that differentiates into visceral and parietal endoderm-like cells along with secretion of basement membranes (Coucouvanis and Martin, 1995). Similarly, 2D stem cell models in geometrically confined microfabricated culture wells recapitulate self-organisation of human ESCs, including differentiation of extra-embryonic endoderm cells at the colony periphery (Warmflash et al., 2014; Camacho-Aguilar et al., 2024).

Stable extra-embryonic endoderm (XEN) stem cell lines were first derived *in vitro* from mouse blastocysts (Kunath et al., 2005). These XEN cells more closely resemble parietal rather than visceral endoderm or primitive endoderm, as evidenced by their transcriptome and limited contribution to visceral endoderm in chimeras (Kunath et al., 2005; Brown et al., 2010). However, XEN cells can be differentiated into visceral endoderm-like cells by stimulation of TGFβ or BMP signalling (Kruithof-de Julio et al., 2011; Artus et al., 2012; Paca et al., 2012). Alternative approaches also allow for the conversion of mouse ESCs into XEN cells. Transcription factor overexpression, principally Gata6, in mouse ESCs is sufficient to induce XEN differentiation, even in culture conditions that would otherwise favour PSC maintenance (Fujikura et al., 2002; Shimosato et al., 2007; Wamaitha et al., 2015). The mechanism for induced XEN differentiation is thought to be due to direct repression of pluripotency-associated transcription and simultaneous activation of a XEN-like gene regulatory network (Wamaitha et al., 2015). By contrast, GATA6 overexpression in human ESCs is not sufficient to drive stable XEN cells. This lack of conservation may be due to the need for additional transcription factors, alternative culture media or differences in the starting PSC state. Indeed, Cho et al. (2012) showed that mouse ESCs at an earlier formative or naïve stem cell state can be converted into XEN cells by exposure to the growth factors retinoic acid and activin, while primed mouse epiblast stem cells were refractory to this conversion and underwent differentiation towards alternative lineages. This complements earlier work in embryoid bodies showing the importance of retinoic acid and Gata6 in extra-embryonic endoderm differentiation (Capo-chichi et al., 2005).

### SCBEMs

Integrated SCBEMs attempt to recapitulate all lineages of the mammalian embryo. In the preimplantation context, ‘blastoids’ have been generated from mouse (Rivron et al., 2018; Sozen et al., 2019; Li et al., 2019b; Jorgensen et al., 2025) and human (Yu et al., 2021; Liu et al., 2021; Yanagida et al., 2021; Kagawa et al., 2022; Yu et al., 2023; Karvas et al., 2023) PSCs. However, in both species, primitive endoderm differentiation remains a challenge (Rivron et al., 2018; Zhao et al., 2024). Efforts to improve the composition of primitive endoderm in SCBEMs have included changes in medium composition and exogenous expression of transcription factors (Sozen et al., 2019; Li et al., 2019b; Jorgensen et al., 2025). Yet the primitive endoderm in SCBEMs remains largely underdeveloped and not well represented, and therefore requires improvement.

In 2022, two groups reported the generation of SCBEMs displaying mouse developmental hallmarks up to embryonic day 8.5, including AVE-like cells that seemed to regulate primitive streak formation and gastrulation (Amadei et al., 2022; Tarazi et al., 2022). Both strategies relied on exogenous expression of Gata4 for extra-embryonic endoderm-like differentiation (Amadei et al., 2022; Tarazi et al., 2022). Given the ease of genetic manipulation, these models may shed more light on stages of mammalian development that are largely poorly characterised, including the development of AVE-like cells (Xiang et al., 2019; Molè et al., 2021). This demonstrates a remarkable ability of stem cells to model complex aspects of development, although the efficiency of reaching these stages is currently limited. In particular, more work is needed to improve the contribution of the extra-embryonic endoderm to these models.

Multiple human SCBEMs of post-implantation development have been established. One strategy relies on the assembly of embryonic and extra-embryonic cell types together, after differentiation by either exogenous expression of molecular markers or exposure to small molecules (Weatherbee et al., 2023; Oldak et al., 2023; Ai et al., 2023; Hislop et al., 2024; Oura et al., 2025). Alternatively, human SCBEMs have been established by culture conditions that promote self-assembly during induced PSC reprogramming or starting from PSCs (Pedroza et al., 2023; Liu et al., 2023; Chen et al., 2025). Despite the different strategies used, all groups report patterning of blastocyst-like structures and molecular signatures of post-implantation development. What is not yet clear is how these models can more consistently form extra-embryonic endoderm-like cells and undergo efficient and robust morphogenesis. If the technical challenges can be overcome, then these models would allow for scalable functional studies and drug screening that would complement direct studies of mammalian embryos.

Implantation models offer another approach to understand extra-embryonic endoderm formation *in vitro* (Ojosnegros et al., 2021; Zhai et al., 2022; Liu et al., 2025). These primarily utilise blastoids and human blastocysts to model implantation onto a plastic surface, extracellular matrix or endometrial cell culture (Shahbazi et al., 2016; Deglincerti et al., 2016; Xiang et al., 2019; Rawlings et al., 2021; Kagawa et al., 2022; Karvas et al., 2023; Shibata et al., 2024; Molè et al., 2026). Recently, several groups reported remarkable development in extended culture of human embryos and SCBEMs up to the equivalent of 14 dpf in endometrial co-cultures. These were used to model aspects of post-implantation morphogenesis and recurrent implantation failure. Primary yolk sac-like cavities were observed upon implantation of both blastoids and blastocysts, although efficiency was either low or not reported (Li et al., 2026; Molè et al., 2026). Strikingly, human embryo co-culture with 3D endometrial organoids led to sophisticated embryonic morphogenesis, including some aspects of yolk sac formation (Song et al., 2025). Nonetheless, more work is needed to increase the robustness and efficiency of transformation from a hypoblast to primary yolk sac structure in these models. Moreover, while endometrial models contain gland and stromal cells, they lack vasculature and immune (NK) cells, which are known to be important for implantation, and a physiological niche for continued development. As improvements are made to fully recapitulate the maternal endometrial environment, the modelling of the early yolk sac is likely to improve and will provide opportunities to elucidate fundamental aspects of the biology of this tissue, including phenomena unique to primates (Ross and Boroviak, 2020).

### *In vitro* models of the yolk sac

Despite evidence indicating roles in embryo nutrition and primitive haematopoiesis during early development, the mammalian yolk sac remains largely understudied (Cindrova-Davies et al., 2017; Ross and Boroviak, 2020; Burton et al., 2025). One strategy for the study of the yolk sac consists of organoid models, derived by *in vitro* culture of primary tissue. Organoids have the potential to recapitulate the organisation and cell heterogeneity of the original tissue, allowing the study of tissue physiology. So far, yolk sac organoids have been generated from canine, bovine, porcine and mouse yolk sac samples (Pereira et al., 2023; Burton et al., 2025). However, ethical constraints, together with the tissue's sensitivity, limit access to human primary tissue at this stage (Burton et al., 2025).

Instead, stem cell models emerge as a scalable alternative for the study of the human yolk sac. Beyond providing biological insights, stem cell models of the yolk sac could be used in the production of blood cells for therapeutic purposes (Atkins et al., 2022; Tamaoki et al., 2023; Hislop et al., 2024). Haematopoiesis has been so far recapitulated in multiple models (Atkins et al., 2022; Tamaoki et al., 2023; Oldak et al., 2023; Liu et al., 2023; Hislop et al., 2024). Moreover, simultaneous incorporation of embryonic cells has allowed the study of cell–cell communication and tissue patterning (Mackinlay et al., 2021; Oldak et al., 2023; Liu et al., 2023; Hislop et al., 2024). Further development of these models could also be used to understand how endometrial histotrophic factors are transported across the yolk sac to maintain a consistent nutrient supply for embryo development. These processes could only be accessible to study in these models, since the secondary yolk sac develops after the 14-day limit to culture human embryos *in vitro*, and therefore is inaccessible using the implantation models discussed above. This demonstrates the importance of developing multiple methodologies to study progressive stages of yolk sac development.

### Limitations and future directions of *in vitro* models of extra-embryonic endoderm

Ground-truth benchmarking of the human yolk sac is needed to serve as a reference for *in vitro* models. Although some single-cell RNA-sequencing datasets are available for human extra-embryonic endoderm, not every time point is covered (Goh et al., 2023). There are several stages in the development of the yolk sac in which the underlying mechanisms are unclear. This includes the transition from the primate primary to secondary yolk sac, the differentiation of visceral and parietal endoderm, and distinctions between extra-embryonic and definitive endoderm. Single-cell multi-omic analysis of these time points is needed; however, access to these stages is, of course, incredibly rare. Implantation models using human embryos may provide an opportunity to make some of these insights. The combination of robust assays of *in vivo* samples and *in vitro* models would allow for more complete characterisation and understanding of this tissue.

A significant challenge in blastoid models is ensuring the efficiency, stability, continual development and proliferation of extra-embryonic endoderm lineages (Li et al., 2019b; Shankar et al., 2021). Furthermore, the dynamic interaction between extra-embryonic mesoderm, trophoblast, endometrium and the extra-embryonic endoderm to form primitive blood islands and initiate haematopoiesis and vascularisation has not been comprehensively modelled. The establishment of culture conditions in which multiple cell types can develop faithfully, robustly and reproducibly will be an important challenge to overcome (Chandrasekaran and Li., 2024).

## Conclusions and outlook

The evolution of extra-embryonic endoderm is a powerful lens through which to understand the evolution of embryo development. Despite its conservation over 500 million years of vertebrate evolution, it has undergone tremendous changes in form and function. Fish and birds retain a syncytial yolk sac endoderm, whereas reptiles demonstrate complete cellularisation of the yolk from the initial yolk sac. Further, mammals and birds have potentially independently evolved unique AVE/hypoblast signalling functions that regulate the start of gastrulation. The yolk sac is the first organ to fully form in many species and, therefore, acts as a window to understand a vast number of roles, including haematopoiesis and axis formation, that all occur in the yolk sac before organogenesis.

Improving our understanding of extra-embryonic endoderm will be important for tackling challenges in fertility. Defective primitive endoderm development is most common in embryos with the least implantation potential (Chousal et al., 2024). Moreover, an analysis of ultrasounds from over 700 pregnancies has shown that abnormal growth patterns of the yolk sac serve as the strongest early predictor of miscarriage (Detti et al., 2020). The human yolk sac is a transient organ, undergoing its entire lifecycle within the first trimester of pregnancy, making it extremely rare to obtain samples of this tissue and incredibly challenging to study its development *in vivo*. Nevertheless, continuous advancements in embryo and stem cell models of extra-embryonic endoderm provide a promising method to study the molecular biology and regulatory mechanisms underlying yolk sac development.
